# Cardiopulmonary factors affecting 6-min walk distance in patients with idiopathic inflammatory myopathies

**DOI:** 10.1007/s00296-018-4050-0

**Published:** 2018-05-14

**Authors:** Naoki Mugii, Fujiko Someya

**Affiliations:** 1 Division of Health Science, Kanazawa University Graduate School of Medical Science, Kanazawa, Japan; 2 School of Health Sciences, Kanazawa University, Kodatsuno 5-11-80, Kanazawa, 920-0942 Japan

**Keywords:** Polymyositis, Dermatomyositis, Exercise capacity, Pulmonary function test, Hemodynamic response, Interstitial lung disease

## Abstract

Idiopathic inflammatory myopathies involve skeletal muscles and can be associated with interstitial lung disease and/or heart dysfunction, which may reduce exercise capacity. We aimed to clarify cardiopulmonary factors affecting the 6-min walk distance in patients who were able to walk without leg pain or fatigue. Twenty-three patients with inactive adult idiopathic inflammatory myopathies, and 18 age- and gender-matched healthy controls were evaluated for hemodynamic responses using noninvasive impedance cardiography during the 6-min walk test. The patients were also examined by the pulmonary function test for forced vital capacity and diffusing capacity for carbon monoxide (DLCO), and by echocardiography for left ventricular ejection fraction and right ventricular systolic pressure. Interstitial lung disease was diagnosed in 19 patients using high-resolution computed tomography. There was no difference in 6-min walk distance or cardiac output after walking between the patients and healthy controls. However, stroke volume during the 6-min walk test was significantly lower in the patients than in healthy controls, suggesting malfunction in the heart. Moreover, the increased heart rate matched the cardiac output. Spearman’s correlation analysis demonstrated a correlation between 6-min walk distance and stroke volume, cardiac output after walking and DLCO, but not left ventricular ejection fraction or right ventricular systolic pressure, as this study lacked the patients with pulmonary hypertension. In conclusion, impaired DLCO due to interstitial lung disease was suggested to be a fundamental parameter affecting exercise capacity, in addition to heart involvement, in patients with idiopathic inflammatory myopathies.

## Introduction

Interstitial lung disease is a common extra-muscular complication of idiopathic inflammatory myopathies, and low-diffusing capacity for carbon monoxide (DLCO) is known to increase the risk of mortality [1, 2]. A recent study demonstrated that recovery of skeletal muscle strength and reduced creatine kinase levels by medication for idiopathic inflammatory myopathies may improve forced vital capacity (FVC) due to muscular strength reinforcement; however, DLCO and interstitial infiltrates on CT were not ameliorated in most cases [3]. In general, distance during the 6-min walk test (6MWT) correlates with FVC and DLCO in patients with idiopathic pulmonary fibrosis [4]. Therefore, we hypothesized that accompanying interstitial lung disease may be a factor affecting exercise capacity in patients with idiopathic inflammatory myopathies, and examined which pulmonary function parameters affect exercise capacity.

Heart involvement in idiopathic inflammatory myopathies was also detected by cardiac magnetic resonance tomography [5] or echocardiography [6], and these findings may lead to therapeutic treatment for the cause of morbidity [7]. An increased risk of myocardial infarction or conduction abnormalities was reported in patients with idiopathic inflammatory myopathies [8]. Moreover, left ventricular diastolic dysfunction [6] and reduced left ventricular ejection fractions (EF) [5] were observed in patients regardless of the presence of myalgia, paresis, and exhaustion. Few studies have demonstrated the relationship between cardiac functions and exercise capacity in patients with idiopathic inflammatory myopathies. It may be difficult to clarify cardiac parameters likely associated with exercise capacity using conventional echocardiography; however, when cardiac impairment was assessed by global longitudinal strain measurement, subclinical heart disease was found in up to 50% of patients [9].

Referring to other estimates for cardiac parameters, recent studies have demonstrated that impedance cardiography with real-time monitoring can noninvasively evaluate hemodynamic responses during the 6MWT [10, 11]. Pulmonary hypertension patients with a normal ejection fraction had a lower stroke volume (SV) and cardiac index (CI) compared with healthy controls during walking [10], and patients with chronic obstructive disease exhibited slower responses to increasing cardiac output (CO) [11]. Moreover, the maximal CO in patients with pulmonary hypertension was observed during the 6MWT by the inert gas rebreathing method [12]. Thus, these noninvasive methods may be available for evaluation of hemodynamic responses during walking to examine the relation between exercise capacity and heart involvement in patients with idiopathic inflammatory myopathies.

In the present study, we investigated lung and cardiac function parameters related to limited exercise capacity in patients with idiopathic inflammatory myopathies. For cardiac function, we compared measurements obtained from patients and age- and gender-matched healthy controls during the 6MWT using noninvasive impedance cardiography.

## Methods

Patients receiving medication for idiopathic inflammatory myopathies were recruited among patients referred to the Rehabilitation Division of Kanazawa University Hospital between November 2014 and September 2017 for consultation on further treatments followed by treatment with glucocorticoids at the acute phase. They received muscular training, pulmonary rehabilitation, and education about activities of daily living. Exclusion criteria for this study were juvenile dermatomyositis, overlap with other connective tissue diseases, muscle weakness in the extremities of less than 4 on manual muscle testing, leg discomfort or pain during the 6MWT, and active myositis, i.e., period of increased serum creatine kinase. In total, 23 patients were assigned to this study (Table 1). According to the criteria of Bohan and Peter or Sontheimer’s criteria, 12 of them were diagnosed as dermatomyositis, 10 as clinically amyopathic dermatomyositis, and 1 as polymyositis. On autoantibody analysis, seven had anti-ARS (anti-Jo-1, PL-7, EJ, and OJ), ten had anti-MDA5, two had anti-SRP, three had anti-TIF1-γ, and one was unknown. Interstitial lung disease was diagnosed in 19 patients by specialists of respiratory medicine using high-resolution computed tomography, mainly with the presence of ground-glass opacities. The study was approved by the human ethics committee of Kanazawa University, conforming to the provisions of the Declaration of Helsinki. Written informed consent for the study was obtained from all patients and 18 healthy controls before the performance of the 6MWT.

**Table 1:** Characteristics of subjects

	Healthy controls (*n* = 18)	Myopathy patients (*n* = 23)	*p*
Gender (f/m)	12/6	13/10	0.51
Age (years)	62 (21; 74)	60 (27; 80)	0.81
Height (cm)	160 (147; 179)	159 (152; 177)	0.91
Weight (kg)	57 (37; 73)	57 (41; 72)	0.74
Body mass index	22 (17; 25)	21 (16; 28)	0.54
6MWT distance (m)	530 (430; 743)	516 (261; 669)	0.28
FVC (% prediction)	–	97.1 (61.3; 138.3)	
DLCO (% prediction)	–	60.8 (37.8; 88.1)	
EF (%)	–	70 (44; 79)	
RVSP (mmHg)	–	26 (12; 35)	
At rest			
Stroke volume (mL)	69.7 (51.3; 95.5)	57.6 (20.7; 92.2)	0.002
Heart rate (beats/min)	75 (57; 105)	85 (64; 117)	0.04
Cardiac output (L)	5.5 (3.5; 7.7)	4.6 (1.7; 8.0)	0.03
Cardiac index	3.7 (1.9; 4.5)	2.7 (1.2; 4.9)	0.02
At 6 min of 6MWT			
Stroke volume (mL)	105.8 (80.1; 136.4)	76.6 (16.5; 122.0)	0.002
Heart rate (beats/min)	118 (84; 161)	134 (89; 197)	0.18
Cardiac output (L)	12.2 (8.2; 17.1)	9.8 (1.5; 20.1)	0.04
Cardiac index	7.8 (5.2; 10.6)	6.2 (1.1; 12.6)	0.04

Values are median (min; max). FVC, DLCO, EF, and RVSP were not measured in healthy controls

*6MWT* 6-min walk test, *FVC* forced vital capacity, *DLCO* diffusing capacity for carbon monoxide, *EF* ejection fraction, *RVSP* right ventricular systolic pressure

**Fig. 1:**
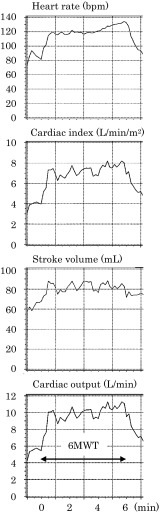
Hemodynamic responses recorded by noninvasive impedance cardiography during the 6-min walk test (6MWT) in a patient

Hemodynamic responses during the 6MWT were measured using the PhysioFlow Q-Link (Manatec Biomedical, France), which weighed 200 g and was wearable. Six disposable electrodes (BlueSensor SP, Ambu, Denmark) connecting to the equipment were placed around the chest for impedance cardiography, as previously described [10]. The 6MWT was performed following the Guidelines of the American Thoracic Society in a 30-m corridor [13]. SV, heart rate, CO, and CI were recorded at rest for autocalibration for 30 s before the 6MWT. Subsequently, the data were automatically averaged every 10 s during the 6MWT, and values at 6 min of walking were collected for analyses (Fig. 1).

The clinical data of percentages of predicted FVC and DLCO (FVC% and DLCO%) from pulmonary function tests, and left ventricular EF and right ventricular systolic pressure (RVSP) from echocardiography were collected by reviewing the medical records of the patients. These evaluations were not performed for healthy controls, as we expected the values to be normal.

## Statistics

Clinical characteristics were compared between the patients and healthy controls using the Mann-Whitney test for numerical data, and chi-square test for gender distribution. The relation between SV at rest and that at 6 min was examined using Spearman’s correlation coefficients for all subjects. Correlation analyses were performed for 6MWT distance and FVC%, DLCO%, EF, RVSP, SV, and CO at rest and at 6 min. JMP 11.0 (SAS Institute Inc., Cary, NC) was used for statistical analysis. A *p* value of < 0.05 was considered significant.

## Results

**Fig. 2:**
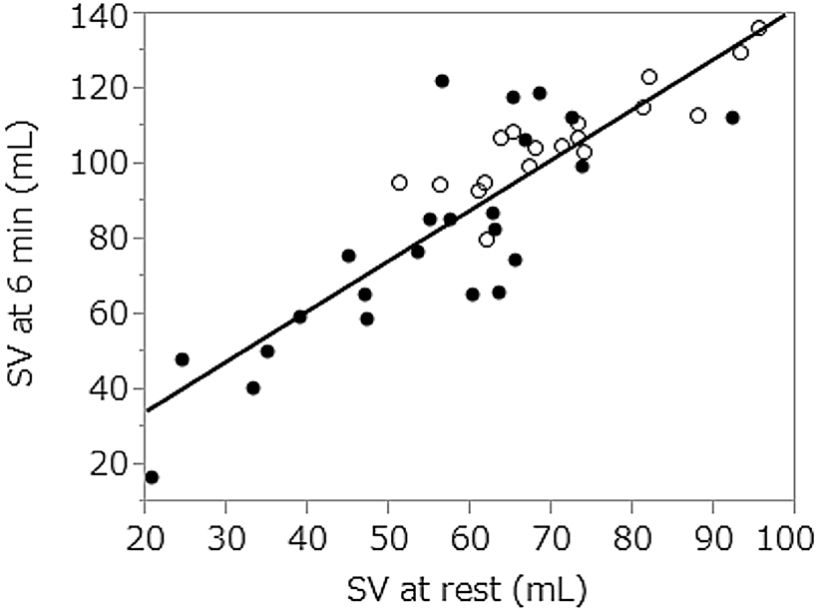
Relationship between stroke volume (SV) at rest and at 6 min during the 6-min walk test. Open circles represent healthy controls and closed circles represent patients with idiopathic inflammatory myopathies. *ρ* = 0.82, *p* < 0.0001

There was no difference in gender distribution, age, or anthropometric measurements between patients and healthy controls. The median DLCO% was low at 60.8% in patients, and seemed to be poorer than the median FVC%, which was 97.1% (Table 1). The EF in patients was not impaired at 70%, except in two patients showing EF < 60%. RVSP reflects pulmonary artery systolic pressure and > approximately 35 mmHg indicates pulmonary hypertension [14], and there were no patients with high RVSP in this study. The SV, CO, and CI at rest and at 6 min were significantly lower in patients than in healthy controls. Patient heart rates were high, but there was no significant difference between patients and healthy controls at 6 min. The 6MWT distance was shorter for patients, but the difference was not significance. A strong correlation between SV at rest and that at 6 min was observed in all subjects (*ρ* = 0.82, *p* < 0.0001) (Fig. 2).

In the healthy controls, the 6MWT distance was correlated with CO at 6 min, but not with SV (Table 2). On the other hand, there was a significant correlation between 6MWT distance and DLCO%, SV and CO at 6 min in the patients. There was no correlation between 6MWT distance and EF or RVSP.

**Table 2:** Correlation between 6-min walk distance and cardiopulmonary parameters using Spearman’s correlation analysis

Parameters	Healthy controls		Myopathy patients	
	*ρ*	*p*	*ρ*	*p*
FVC (% prediction)	–	–	0.25	0.30
DLCO (% prediction)	–	–	0.66	0.007
EF (%)	–	–	− 0.10	0.66
RVSP (mmHg)	–	–	− 0.28	0.27
Stroke volume (mL), at rest	< 0.01	0.99	0.38	0.07
Cardiac output (L), at rest	0.10	0.68	0.33	0.12
Stroke volume (mL), at 6 min on 6MWT	0.18	0.48	0.61	0.002
Cardiac output (L), at 6 min on 6MWT	0.65	0.004	0.62	0.002

FVC, DLCO, EF, and RVSP were not measured in healthy controls

*6MWT* 6-min walk test, *FVC* forced vital capacity, *DLCO* diffusing capacity for carbon monoxide, *EF* ejection fraction, *RVSP* right ventricular systolic pressure

## Discussion

This is the first study to evaluate exercise capacity in patients with idiopathic inflammatory myopathies in relation to lung and heart function parameters. EF is a widely used parameter representing cardiac function [15], and if patients with idiopathic inflammatory myopathies have reduced EF (< 60%), late gadolinium enhancement by cardiac magnetic resonance tomography is observed [5]. In this study, EF was normal in most patients and there was no correlation with 6MWT distance, whereas the SV was significantly lower than that in healthy controls. Therefore, EF was unable to be used to estimate the exercise capacity in our cohort. Pulmonary arterial hypertension would affect 6MWT distance [16], but we could not present the relationship because no patient showed high RVSP. Previously, the association of pulmonary arterial hypertension and idiopathic inflammatory myopathies has rarely been reported [17].

SV at 6 min in healthy controls was not correlated with 6MWT distance. It was previously reported that in healthy subjects, heart rate response contributed to CO or oxygen uptake rather than the SV response during exercise [18]. Additionally, varying response patterns in SV to exercise were previously observed in healthy subjects, but the mechanism was not clarified [19]. On the other hand, in this study, SV at rest was highly correlated with SV at 6 min in all subjects, and little variation in SV was noted in response to the 6MWT. Therefore, the significant deterioration in SV at rest in patients implied proportionally low SV during walking, which affected 6MWT distance. Impaired SV at rest or at 6 min suggests malfunction in the heart not detected by EF.

CO is known to be correlated with oxygen uptake during exercise in healthy subjects [20], and CO at 6 min was also correlated with 6MWT distance in healthy controls and patients. As CO and CI at 6 min were compensated for by the increased heart rate despite lower SV in patients, the difference in 6MWT distance between patients and healthy controls was unclear, which suggests that CO compensation during exercise influences exercise capacity.

Another variable affecting 6MWT distance was DLCO% in patients, and this result was considered to confirm our hypothesis that accompanying interstitial lung disease affects the exercise capacity in patients. As interstitial lung disease was diagnosed in 82% (19 of 23 patients) of the patients in this study, it may be the principal manifestation affecting exercise capacity in our cohort. A significant relationship between DLCO and exercise capacity was previously confirmed in patients with idiopathic pulmonary fibrosis [4, 21]. In addition, DLCO better represented impairment of pulmonary gas exchange than other parameters such as PaO_2_, alveolar–arterial oxygen pressure difference, and pulmonary capillary blood volume [22, 23].

FVC is another parameter on the pulmonary function test, but it was not related with 6MWT distance in the patients. For this reason, the value of FVC%, which had a median value of 97.1%, was normal compared with DLCO%, which was 60.8%. As the patients had inactive myositis, FVC may have been recovered [3] when we evaluated them. If the patients were more impaired, the exercise capacity may have been affected, as reported for patients with idiopathic pulmonary fibrosis [4].

Regarding other factors for exercise capacity, a previous study failed to find a correlation between serum creatine kinase and peak oxygen uptake or peak isometric torque in patients with inactive idiopathic inflammatory myopathies [24]. In addition, glucocorticoids and other medications may cause muscle weakness that lasts for several weeks or years [25]. Thus, many factors should be taken into consideration when evaluating the exercise capacity in patients with idiopathic inflammatory myopathies. However, we set the exclusion criteria for this study as muscle weakness in the extremities of less than 4 on manual muscle testing and leg discomfort or pain during the 6MWT to clarify the effects of cardiopulmonary functions on exercise capacity by excluding muscle factors. If all factors, such as muscle fatigue during active myositis, were included in the analysis, a different result may have been obtained.

The limitation of this study was the extraction condition of patients mentioned above. These results cannot be extended to patients who are unable to walk sufficiently. Our aim was to clarify the effects of lung and heart involvement on exercise capacity in patients with a certain condition. We demonstrated the influence of pulmonary factors in addition to cardiac factors on exercise capacity in these patients, and pulmonary rehabilitation is recommended to improve their functional status and quality of life [26]. However, idiopathic inflammatory myopathies generally involve skeletal muscle, and the relationship between muscle symptoms and exercise capacity should be examined in the future.

## Conclusion

Among the manifestations of idiopathic inflammatory myopathies, we evaluated deterioration in the lung and the heart in relation to exercise capacity using the 6MWT. The SV during the 6MWT was impaired in patients compared with healthy controls, and the parameters influencing 6MWT distance were SV and CO at 6 min and DLCO%. Interstitial lung disease may be a factor-limiting exercise capacity in patients with idiopathic inflammatory myopathies who can walk.
